# Childhood adversity, perceived social support, and depressive symptoms among pre-abortion Chinese women

**DOI:** 10.1186/s12978-024-01811-3

**Published:** 2024-05-22

**Authors:** Shuyan Yang, Yini Wang, Boye Fang, Bei Chen, Peishan Chen, Lili Xie, Zilu Zhong, Gengzhen Chen

**Affiliations:** 1 Department of Innovative Social Work, City University of Macau, Macau, China; 2 Cancer Hospital of Shantou University Medical College, Shantou, China; 3 School of Sociology and Anthropology, Sun Yat-Sen University China, Guangzhou, China; 4 Institute of Environment and Ecology, Tsinghua Shenzhen International Graduate School, Shenzhen, China; 5 Department of Obstetrics and Gynecology, Shantou University Medical College, Shantou, China; 6 Teachers College, Columbia University, New York, USA; 7 People’s Hospital of Chenghai, Nantian Road, Chenghai District, Shantou, Guangdong Province China

**Keywords:** Depressive symptoms, Childhood adversity, Sources of social support, Pre-abortion women

## Abstract

**Background:**

Unintended (unwanted) pregnancy is a sexual and reproductive health issue with psychosocial consequences for the individual, their family, and society. However, the relationship between social support and related mental health issues, like depression and the effects of childhood adversity, is poorly studied. This study aims to explore the connections between childhood adversity, perceived social support, and depressive symptoms in pre-abortion women (women who have decided to have an abortion) in a clinical setting, based on the common risk factor approach and social support theory.

**Methods:**

A total of 299 pre-abortion Chinese women 18–45 years were recruited in a hospital in Shantou, China. Hierarchical linear regression analyses were employed to examine the relative effects of childhood adversity and sources of social support on depressive symptoms, controlling for sociodemographic influences*.*

**Results:**

The results show that 37.2 percent of participants reported at least one adverse experience in childhood. More than half of the respondents were at risk for depression. Results of regression analysis showed that childhood adversities were negatively associated with depressive symptoms before sources of social support were entered into the model. However, when the sources of perceived social support were added, the effect of childhood adversity was not significant. Perceived social support explained the additional 15 percent variance in depressive symptoms. Additionally, being married (β = -.12, *p* < .05) and number of siblings (β = .13, *p* < .05) were significantly related to depressive symptoms.

**Discussion:**

Pre-abortion women are at risk of mental health problems. Peer and familial social supports can alleviate the influence of childhood adversity on depression among pre-abortion Chinese women. Strengthening the role of various sources of social support can help to improve the mental health conditions of pre-abortion women.

## Introduction

Unintended (unwanted) pregnancy is a sexual and reproductive health issue with long-term psychosocial consequences for the individual and society [1]. However, the effects of abortion on women's mental health after abortion surgery remain a matter of debate in current scientific inquiries [2, 3]. A recent study has suggested that a deeper insight into the mental health of pre-abortion women—namely, women who have decided to have an abortion—could reveal more about their mental health challenges after the abortion [4]. Empirical studies have documented that a substantial proportion of pre-abortion women are ambivalent and uncertain about their course of action, and they may be prone to poor psychological outcomes [5], or be unlikely to consult with their regular gynecologic care provider about their abortion decision [6]. More assessment of those women's mental health conditions at the pre-abortion stage will help healthcare professionals to identify possible risks and provide quality care services both before and after abortion surgery.

### Mental health issues in women with unintended pregnancy

Depression is a severe mental disorder with a chronic course of emotional dysregulation and depressive cognition that affects more than 300 million people [7]. The severity of depression is widely used as an important indicator when assessing individuals’ mental health [8]. Emerging evidence from the United States and Europe has shown that women with an unwanted pregnancy who are at a pre-abortion stage demonstrate higher levels of depressive symptoms and stress during the pre-abortion and post-abortion periods [8]. A better understanding of what influences pre-abortion mental health may help these women cope with the stress more effectively and have a better post-abortion adaptation outcome.

The common risk factor approach argues that a series of common risk factors/confounding influences should be examined when comparing the psychological outcomes for a woman having an abortion [9]. These factors include pre-existing psychiatric problems, such as childhood adversity, disadvantaged social economic status, and so forth. Adverse experiences/events in childhood may have long-term impacts on women’s physical and mental health [10]. Von Cheong et al. [11] noted that early life adversities have a long-term enduring effect on the person’s response to other life events.

Adverse childhood experiences (ACEs) describe any acts of commission or omission by a parent or other caregiver that result in actual and potential harm or the threat of harm to a child at or before the age of 18 years [11]. ACEs include all types of abuse and neglect as well as household dysfunction, such as parental divorce, substance abuse, mental illness, and domestic violence. Existing studies have shown that childhood adversities are associated with depressive symptoms in adulthood, at working age, and even into older age [10, 11]. However, there is limited research investigating the impacts of childhood adversity on psychological health among women before abortion surgery. Only a handful of studies have reported that a personal experience of childhood adversity is associated with an increased likelihood of a subsequent abortion or multiple abortions [12] and both elevated anxiety and stress [4].

Social support refers here to a web of social relationships that can help individuals cope with stressful events more effectively [13]. The social support theory posits that multiple sources of social support are effective in buffering against adverse life experiences and depression [14]. Additionally, empirical research has conceptualized perceived social support from various networks of support, including families, friends, and significant others [15]. A study of 404 pregnant Italian women found that those with higher levels of social support or a positive experience during pregnancy were less likely to experience antenatal depression [16]. For women have decided to have an abortion, their partner, parents, and friends have long been regarded as important sources of care and support [17], especially when women have insufficient financial resources or limited access to specific knowledge. Yet it remains unclear whether and to what extent these various forms of social support affect depressive symptoms among the target women. Therefore, assessing the social support resources, such as the networks of pre-abortion women, is critical to enhancing their mental health: identifying factors that shape the processing of events following exposure to ACEs may be a valuable tool to develop tailored interventions aimed at preventing or mitigating the long-term mental health consequences of ACEs among these women.

Abortion in Chinese women is largely shaped by distinctive cultural factors and sociopolitical circumstances. Historically, Chinese people have mainly adopted conservative views in matters of sexual and reproductive health. In general, traditional values and norms of sexuality restrict a woman’s assertiveness in sexual activities [18]. If a girl internalizes this gender role, she may either lack contraceptive knowledge or not feel assertive enough to ask boys to use condoms, often leading to an unplanned pregnancy [8]. More importantly, as a typical patriarchal society, traditional Chinese collectivist values assert that for an unmarried woman to deal with an unwanted pregnancy by seeking an abortion rather than entering a marriage is particularly disgraceful and causes her family to ‘lose face’.

The available empirical studies of unintended pregnancy in Chinese contexts have either sampled largely unmarried university/college students or migrant factory workers [8]. Samples of abortion-seeking women drawn from clinical settings before induced abortion are few. Although one study has used clinical samples to investigate repeated abortions among young women [19], it has a biomedical focus, thus leaving psychological health and psychosocial factors relatively understudied. To facilitate the screening process and provide customized counseling/care to women in the pre-abortion stage, a study of depressive symptoms among those women in a clinical setting is imperative.

### The present study

The present study was guided by the common risk factor approach and social support theory. The common risk factor approach argues that a series of common risk factors should be examined when comparing pre-abortion women's psychological outcomes. Social support theory claims that different social support sources might mitigate the possible negative effects of adverse life experiences and depression in the target population. Hence, the study aims to investigate the relationship between childhood adversities, social support, and depression in pre-abortion Chinese women. The specific objectives are: 1) to explore the prevalence of childhood adversities and depression in pre-abortion Chinese women using a clinical sample; 2) to examine whether childhood adversity and sources of social support (i.e., family support, peer support, and support from significant others) are associated with depressive symptoms; and 3) to adjust for sociodemographic covariates in the analysis of the association between childhood adversity, social support, and depression. The hypotheses are: 1) a sizable proportion of participants who experience adverse events during childhood are at risk of depression; and 2) the number of childhood adversities and sources of social support (i.e., significant others, peer support, and family support) are independently associated with depressive symptoms in pre-abortion Chinese women, adjusting for sociodemographic covariates.

## Methods

### Participants

The research was conducted in Shantou, Guangdong province, People’s Republic of China, from April 2019 to October 2019. Ethical approval (for human research) was obtained from the Research Committee of the authors’ affiliated university. The participants were informed of the objectives of the study by a survey interviewer. Meanwhile, a brief instruction stating the scope and purposes of the study was distributed to potential participants. Written consent forms were obtained from participants before conducting the survey interviews.

Inclusion criteria for participants in the proposed study were: 1) women between 18 and 45 years of age seeking an abortion at the hospital; 2) fluent in Mandarin or the local dialect and capable of answering the interview questions; 3) a local residence; and 4) voluntary participants. Participants were excluded in cases of fetal anomaly or if rape was the reason for seeking a termination of pregnancy.

In total, 301 participants were recruited from an A-grade hospital in Shantou. After deleting unengaged responses, 299 participants were included in the final analysis. The A-grade hospital was the First-Class Tertiary Hospital, which has been widely used in mainland China’s hospital evaluation system due to its social reputation and higher quality of services [20]. Participants were asked by the front desk staff at the hospital if they were willing to participate in a study on women’s reproductive health. If so, trained interviewers guided the woman to a private room at the Office of Reproductive Health to conduct an interview or administer a questionnaire; participants could choose to self-administer the survey. One student with a medical background was trained before data collection and collected survey data. With the consent of the participants, the face-to-face survey was conducted with the participant by the interviewer.

### Instrument

#### Depressive symptoms

Participants’ sense of anxiety and depression was assessed using the Hospital Anxiety and Depression Scale (HADS), which is a questionnaire that measures anxiety and depression in a general medical population of patients. The Chinese translation of the HADS demonstrates good reliability [8]. In this 14-item scale, the subset of depression comprised seven items. The depression subscale had a potential total score range of 0 to 21, a higher score indicating higher levels of depressive symptoms. The Cronbach’s alpha of depressive symptoms for this study was 0.86, which was satisfactory.

#### Childhood adversity

The situation of participants’ adverse childhood experiences was assessed using the 10-item ACE scale [8]. The Chinese version of the ACE scale has also been proven to be valid and reliable [21]. The ACE scale was divided into three types: abuse (psychological, physical, and sexual abuse); neglect (psychological and physical neglect); and household dysfunction (parental separation/divorce, mother treated violently, household substance abuse, household mental illness, incarcerated relative). Respondents were asked to report whether or not they had experienced abuse, neglect, and/or household dysfunction in childhood [11]. The total score had a potential range of 0 to 10, a higher score representing more adverse experiences in childhood. The reliability coefficient of this measure was 0.88.

#### Perceived social support

The Multidimensional Scale of Perceived Social Support (MSPSS) was applied to assess participants’ perceived degree of social support from various social support sources [22]. The scale consists of 12 items with three dimensions, including significant others, family, and peers. Respondents were asked to rate each item using a five-point Likert scale. In this study, a higher score indicated a higher level of social support. The internal consistencies of the three dimensions—namely significant others, family, and peer support—were 0.88, 0.90, and 0.89, respectively.

#### Sociodemographic variables

Participants was asked to provide sociodemographic information, such as their age, education level (1 = primary level or below, 2 = junior high school level, 3 = high school or vocational school level, 4 = college or bachelor degree, 5 = master’s degree or above), marital status (0 = unmarried or 1 = married), number of siblings, and household income (1 =  < 2,500 yuan, 2 = 2,500 to 5,000 yuan, 3 = 5,000 to 10,000 yuan, 4 = 15,000 to 20,000 yuan, 5 = 20,000 yuan above). Sociodemographic variables were included in this study as covariates.

### Data analysis

The current study aimed to examine patterns and predictive factors of depressive symptoms. Firstly, the results descriptive statistics were provided. Secondly, correlation analyses were conducted between the main variables studied, such as demographics, childhood adversity, social support, and depressive symptoms. Thirdly, a series of hierarchical multiple regressions were used to determine the relative impact of childhood adversity and sources of social support on depressive symptoms among the sample women, adjusting for sociodemographic covariates. Since missing values account for less than 5 percent of the total sample, we employed the listwise function in SPSS in regression analyses. In addition, all the variance inflation factor (VIF) estimates in regression analyses were below 10, which indicates freedom from multicollinearity issues. In the final model, we examined the standard regression coefficients to determine significant predictors of depressive symptoms. Additionally, we examined the increase in the adjusted *R*^*2*^ value when adding childhood adversity and social supports to determine which predictors contributed a significant amount of variance to the dependent variable (i.e., depressive symptoms). All the statistical analyses were carried out using SPSS 21.0 [23].

## Results

### Descriptive statistics

Table 1 shows the descriptive statistics of the sample. The ages of the participants varied from 18 to 45 years, with an average 26 years (*M* = 26.00, *SD* = 4.90). Almost half of the respondents (46.6%) had a college or undergraduate degree, while more than a quarter (27.8%) had a high school or vocational diploma. Most of the participants (69.9%) were not married. The majority of the participants had siblings. The participants reported high levels of social support from different sources, with mean scores of 3.85 (range 1–5) for significant others, 3.82 (range 1–5) for family, and 3.80 (range 1–5) for friends. More than half of the respondents (51.4%) were at risk for depression, 27.1 percent having borderline depression (score 8–10) and 24.3 percent having depression (score 11–21).

**Table 1:** Demographic characteristics of participants

Characteristics	N^a^ (%)	Mean (SD^b^)	Range
Age (mean + SD)		26.00 (4.90)	18–45
Education level			
Primary level or below	8 (2.7%)		
Junior high school level	27 (9.0%)		
High school or vocational school level	83 (27.8%)		
College or Bachelor’s degree	139 (46.5%)		
Master’s degree or above	40 (13.4%)		
Marital status			
Married	90 (30.1%)		
Unmarried	209 (69.9%)		
Number of siblings			
None	82 (27.4%)		
One	82 (27.4%)		
Two	81 (27.1%)		
Three or more	40 (13.4%)		
Household income			
< 2,500 yuan	1 (0.3%)		
2,500–5,000 yuan	29 (9.7%)		
5,000–10,000 yuan	118 (39.5%)		
15,000–20,000 yuan	104 (34.8%)		
20,000 yuan and above	44 (14.7%)		
Number of childhood adversities	299 (100%)	1.38 (2.28)	0–10
Social support			
Significant others	299 (100%)	3.85 (0.77)	1–5
Family	299 (100%)	3.82 (0.82)	1–5
Peers	299 (100%)	3.80 (0.80)	1–5
Depressive symptoms	299 (100%)	7.02 (4.19)	0–21

^a^ *N* = 299

^b^ *SD* standard deviation

### Prevalence of adverse childhood experiences

Table 2 presents the prevalence of adverse childhood experiences. In all, 37.2 percent of participants reported experiencing at least one adverse experience at or before the age of 18, while more than 60 percent reported never experiencing any form of childhood adversity. Overall, the most common adverse experience in childhood was abuse (29.1%), followed by neglect (26.8%) and household dysfunction (25.4%).

**Table 2:** Prevalence of adverse childhood experience

Prevalence	N^a^	%
Abuse		29.1%
Any psychological abuse	72	24.1%
Any physical abuse	28	9.4%
Any sexual abuse	38	12.7%
Neglect		26.8%
Any psychological neglect	63	21.1%
Any physical neglect	40	13.4%
Household dysfunction		25.4%
Parental separation/divorce	24	8.0%
Mother was treated violently	56	18.7%
Someone in household was a substance abuser	33	11.0%
Someone in household was anxious or depressed	35	11.7%
Someone in household went to prison	23	7.7%
Number of childhood adversities		
One or two of the above adversities	111	37.2%
Three or four of the above adversities	66	22.2%
Five or more of the above adversities	44	14.8%

*N* = 299

### Correlations among studied variables

Table 3 shows the correlation coefficients between depression and the selected variables. The variables were all correlated with depression in the expected direction. Depression had positive correlations with number of siblings (*r* = 0.29, *p* < 0.01) and ACE (*r* = 0.30, *p* < 0.01), and negative correlations with age (*r* = -0.19, *p* < 0.01), education (*r* = -0.33, *p* < 0.01), being married (*r* = -0.30, *p* < 0.01), and family income (*r* = -0.15, *p* < 0.01). Depression also had negative correlations with the sources of social support, such as family (*r* = -0.49, *p* < 0.01), peers (*r* = -0.55, *p* < 0.01), and significant others (*r* = -0.47, *p* < 0.01).

**Table 3:** Correlations among major variables

		1	2	3	4	5	6	7	8	9	10
1	Age	1.00									
2	Education	0.29**	1.00								
3	Married	0.48**	0.22**	1.00							
4	Number of siblings	-0.09	-0.34**	-0.19**	1.00						
5	Family income	0.14*	0.32**	0.16**	-0.29**	1.00					
6	Number of childhood adversities	-0.08	-0.20**	-0.18**	0.12*	0.00	1.00				
7	Social support from significant others	0.10	0.33**	0.22**	-0.25**	0.19**	-0.31**	1.00			
8	Social support from family	0.16**	0.45**	0.29**	-0.26**	0.24**	-0.47**	0.640**	1.00		
9	Social support from friends	0.10	0.37**	0.26**	-0.24**	0.19**	-0.35**	0.87**	.0.68**	1.00	
10	Depressive symptoms	-0.19**	-0.33**	-0.30**	0.29**	-0.15**	0.30**	-0.47**	-0.49**	-0.55**	1.00

*N* = 299

*p < .05, **p < .01, ***p < .001

### Regression analyses

A series of hierarchical regression analyses was conducted to explore the relative contribution of demographic and psychosocial factors to depressive symptoms. The results are summarized in Table 4. Demographic characteristics, including age, education, marital status, number of siblings, and family income, were entered as Block 1. ACE was entered as Block 2. Sources of social support—namely, significant others, family, and friends—were entered as Block 3. It is shown that the adjusted R^2^ values of depression were increased in Blocks 2 and 3, respectively. The total amount of variance explained by all the predictors was 36 percent of depressive symptoms.

**Table 4:** Result of hierarchical multiple regression analyses (DV: depression)

	Model 1		Model 2		Model 3	
	Beta	t-value	Beta	t-value	Beta	t-value
Age	0.02	0.24	0.00	0.07	-0.05	-0.91
Education	-0.22	-3.64***	-0.18	-3.02**	-0.04	-0.64
Marital status	-0.25	-4.03***	-0.22	-3.49***	-0.12	-2.12*
Number of siblings	0.17	2.96**	0.16	2.80**	0.13	2.42*
Family income	0.03	0.49	0.01	0.17	0.05	0.94
Number of childhood adversities			0.21	3.86***	0.06	1.11
Social support from significant others					0.05	0.52
Social support from family					-0.15	-2.10*
Social support from friends					-0.40	-3.87***
R^2a^	0.18		0.22		0.36	
R^2^ change			0.04***		0.15***	

^a^ *R* ^*2*^ = adjusted R square

^*^ *p* < .05, **p < .01, ***p ≤ .001

Sources of social support, entered in Block 3, explained the most total additional variance for depressive symptoms with an additional 15 percent of the total variance. Number of childhood adversities accounted for 4 percent of the total variance in depressive symptoms in Block 2. Interestingly, in the final model (Block 3), the number of childhood adversities was no longer associated with the presence of depressive symptoms when sources of social support (significant others, family, and friends) were entered into the regression equation.

In the final model, feelings of depression were predicted by social support from family (β = -0.15, *p* < 0.05) and from friends (β = -0.40, *p* ≤ 0.001), being married (β = -0.12, *p* < 0.05), and number of siblings (β = 0.13, *p* < 0.05). The results show that peer support is the best predictor for relieving symptoms of depression. Notably, childhood adversity (β = 0.21, *p* ≤ 0.001) was associated with depressive symptoms in Block 2, but the relationship was not significant in Block 3. We performed supplementary analyses regarding the impact of individuals’ childhood adversity on depressive symptoms using the final model in Table 5. That said, the effects of all types of childhood adversity on depressive symptoms were non-significant after adjusting for sources of social support.

**Table 5:** The effect of individual childhood adversity on depressive symptoms in final model (supplementary analysis)

Individual ACE domain		
	Beta	t-value
Psychological abuse	0.01	0.18
Physical abuse	0.02	0.38
Sexual abuse	0.03	0.68
Psychological neglect	0.05	0.99
Physical neglect	-0.02	-0.45
Parental separation/divorce	0.08	1.60
Mother was treated violently	0.04	0.84
Someone in household was a substance abuser	0.02	0.47
Someone in household was anxious or depressed	0.04	0.90
Someone in household went to prison	0.05	1.12

## Discussion

This study has used a clinical sample to explore the prevalence of childhood adversity and salient factors of depressive symptoms among Chinese pre-abortion women in Shantou, China. To the authors’ knowledge, this is the first research to examine the prevalence of childhood adversity and predictive factors of depressive symptoms in a clinical sample of pre-abortion Chinese women. Our study shows that the mental health of pre-abortion women warrants attention: about 37.2 percent of the sample women reported having at least one adverse experience during childhood, and 51.4 percent were at risk for depression. Nevertheless, sources of social support, namely peer support and family support, buffered the negative effects of ACE and subsequent depressive symptoms among the sample pre-abortion Chinese women. Strengthening pregnancy termination counseling and enhancing social support for these women, both before and after abortion surgery, is important. This study provides valuable information about the mental health conditions of pre-abortion women in the pre-abortion stage.

In general, the mental health state of pre-abortion women warrants attention, as a sizable proportion of participants reported mental health issues. Nearly 40 percent of informants reported at least one ACE in the present study: 29.1 percent, 26.8 percent, and 25.4 percent of participants reported experiencing abuse, neglect, and life events, respectively. The reported rates of childhood adversity in the current research were comparably lower than in a previous study of pre-abortion women in Europe, which revealed that 83 percent of participants reported at least one childhood adversity [4]. More than half of the sample respondents (51.4%) suffered the risk of depression, nearly 30 percent reported borderline depression, and an additional one quarter regarded depression in the pre-abortion stage, which is similar to a study reporting that 58 percent of their sample of pre-abortion women were depressed [17].

Previous studies suggested that women who experienced childhood adversity were more likely to have depressive symptoms when they had an abortion in the Netherlands [4]. However, our results indicated that childhood adversity did not have a significant effect on depressive symptoms when we controlled for sources of perceived social support in the final regression model based on social support theory. Therefore, sources of social support (i.e., support from family and peers) mitigated the negative effects of ACE in these women. Our study emphasizes the importance of social support as the protective factor against depression in our sample of Chinese women who were about to have an abortion. Among the three sources of social support, peer support was the most important. It is possible that these women who wanted to have an abortion may find it easier to discuss sexual and reproductive health issues with their peers than with their families (i.e., parents). Providing quality care is especially important for women with unwanted pregnancies [8]. We recommend that the mental health conditions (i.e., depressive symptoms and childhood adversities) of women who have abortions in hospital should be carefully assessed. Our study’s findings imply that social support from multiple sources, especially support from peers and family, should be part of pre-abortion counseling sessions in reproductive health in Chinese hospitals.

Our results confirm that being married and number of siblings are factors prominently associated with depressive symptoms in the final integrated model. Marriage has long been considered an optimal pathway to the achievement of social inclusion [24]. Our study further confirmed that married women had lower levels of depressive symptoms compared to their unmarried counterparts among women undergoing abortion. In other words, it postulated that marriage may act as a protective factor and that it has potential mental health benefits for women who share the experience of abortion. In general, married couples may plan their preferred number of children before or during their marriage, unlike unmarried couples. It is possible that the married women sampled already had their ideal number of children and therefore sought abortions when they experienced unwanted pregnancies. Furthermore, preference for a son is a deeply rooted traditional Chinese childbearing norm: for example, using a nationally representative survey in China, one study indicated that married women whose youngest living child was a girl were more likely to undergo an induced abortion than their counterparts with the same number of children but whose youngest living child was a boy [25]. Compared to the married women, the unmarried women were at higher risk of depressive symptoms, according to our study. Sexually active and unmarried youths are often at considerable risk of unintended pregnancy and abortion. A woman’s unmarried status has long been an unspoken reason for the decision to seek an abortion. A prior qualitative study suggests that the sociocultural acceptability of premarital sex and lack of sufficient contraception awareness are the main reasons for an increase in abortion in China [26].

In addition, our research findings established a new association between number of siblings and depression, suggesting that the number of siblings is positively associated with increased symptoms of depression. A prior study showed that pre-marital sex and pregnancy are highly stigmatized among family members of young women in India [27]. Likewise, traditional Chinese collectivist norms suggest that sexual activities should be conducted within marriage and for the purpose of procreation [18]. In China, it is not surprising if an unmarried woman has an unwanted pregnancy and seeks an abortion, as it may make her family members feel ashamed in the larger community. Thus, more assistance programs/interventions targeted at pre-abortion women in a clinical context should be launched in China. It is necessary to encourage and support various organizations to provide counseling and intervention, especially for unmarried women undergoing abortion.

### Implications

This study's findings have important research and practice implications. The study showed that a substantial proportion of women in the sample reported early childhood adversity and depression. It is necessary to strengthen pregnancy termination counseling both before and after abortion surgery. Tailor-made interventions should be based on women's pre- and post-abortion worries and demands. Education programs concerning unintended pregnancy in young adults should be introduced in mainland China cities, with special focus on childhood adversity and sources of social support. A tailor-made handbook on pregnancy and abortion for pre-abortion women should be produced for this target population. Women should understand and prepare for the possible physiological and psychological consequences of abortion. Evidence shows that deciding to terminate a pregnancy is challenging for young women with an unwanted pregnancy; some women still struggle even as they enter the clinic [28]. These women are entitled to be in a better position to make an abortion decision. In addition, establishing an online peer support platform could help women positively cope with abortion, as our research has shown that peer support is effective in reducing symptoms of depression. Based on the familial and peer support observed in the current study, training and education on reproductive health should be provided to target women, health care providers, and human service professionals to equip them with knowledge concerning the mental health conditions of women undergoing abortion surgery.

### Limitations

The present study has multiple limitations and caution should be exercised when interpreting its findings. Firstly, given the cross-sectional nature of the study, causality cannot be inferred to all the Chinese pre-abortion women. Secondly, data for this survey are collected based on convenience, and some participants may decline to be interviewed due to the study’s topic. It is possible that pre-abortion women may suffer from more mental health problems before the abortion—for instance, depressive and anxiety symptoms. It is reasonable that some participants may decline our survey interview. Thirdly, variables included in this study were self-reported, and so are subject to recall bias and social desirability. Women may under-report their childhood adversities or depressive symptoms as a result of social norms or cultural expectations. In addition, the depression measure used in this study (HADS-D) was based on a self-reported score instead of a medical diagnosis, which may be subject to bias. Fourthly, as the aim of this study was only to target psychological factors in women in the pre-abortion stage, we did not count how many cases ultimately did not undergo abortion surgery. We highly recommend that future studies investigate the mental health conditions in pre-abortion women and post-abortion women using a longitudinal research design. Finally, some variables that might have a confounding influence on the mental health conditions of pre-abortion women were not included in the data, such as marital relationship, family size, relationships with siblings, and family history of mental illness.

## Conclusion

Nearly 40 percent of pre-abortion women reported having at least one adverse experience during childhood, and more than half of participants were at risk for depression. Sources of social support (i.e., peer support and family support) buffered the negative effects of ACE and subsequent depressive symptoms among pre-abortion Chinese women. Multiple sources of perceived social support should be strengthened to reduce depressive symptoms and early childhood adversities among pre-abortion women.

## Data Availability

The dataset used in this study is available from the authors upon reasonable request.
